# The Clinical Characteristics and Outcomes of Follicular Bronchiolitis in Chinese Adult Patients

**DOI:** 10.1038/s41598-018-25670-8

**Published:** 2018-05-08

**Authors:** Ju Lu, Miao Ma, Qi Zhao, Fanqing Meng, Dongmei Wang, Hourong Cai, Mengshu Cao

**Affiliations:** 1 Department of Respiratory Medicine, Nanjing Drum Tower Hospital, Nanjing University Medical School, Nanjing, China; 2 Department of Respiratory Medicine, Nanjing Drum Tower Hospital, Nanjing Medical University, Nanjing, China; 3 Department of Pathology, Nanjing Drum Tower Hospital, Nanjing University Medical School, Nanjing, China; 4 Department of Radiology, Nanjing Drum Tower Hospital, Nanjing University Medical School, Nanjing, China

## Abstract

Follicular bronchiolitis (FB) is a rare interstitial lung disease (ILD) and has been reported in diverse clinical contexts. Six FB patients demonstrated by surgical lung biopsy (SLB) were reviewed between 2009 and 2017 from Nanjing Drum Tower Hospital in China. The average age of subjects was 42 years old (range: 31–55 years). The clinical symptoms were very mild. The laboratory findings showed elevated Erythrocyte sedimentation rate (ESR) and serum globulin and anemia. Pulmonary function tests were normal in four cases. Five cases had underlying diseases, such as, Sjo¨gren’s syndrome, multi-centric castlemans’ disease, idiopathic pneumonia with autoimmune features and abscess. Five cases presented as interstitial lung disease (ILD) on chest imaging with centrilobular or peribronchiolar nodules, ground glass opacities, interlobular septal thickening, cysts and bronchiectasis. Isolated mass was in one patient. The histopathology suggested the changes of FB in all subjects. Prednisone and/or cyclophosphamide were used in four cases, one did the surgery and the other was clinically monitored. All cases were alive at the end of follow up. The adult patients of FB usually have mild symptoms, ILD and underlying diseases. The definite diagnosis needs SLB. The prognosis is depended on their underlying conditions.

## Introduction

Follicular bronchiolitis (FB) is rare bronchiolar disorder characterized by the development of hyperplastic lymphoid follicles with germinal centers around the small airways^1^. With the enlargement of follicles, the partial bronchial and bronchiolar project into the bronchial lumen. The architecture of the bronchial tree would often be distorted, causing partial bronchial and bronchiolar obstruction. FB is a pathological diagnosis and the precise cause is still unknown. It can be generally classified into primary and secondary form. Secondary FB is usually associated with multiple systemic and pulmonary diseases such as connective tissue disease (CTD) and immunodeficiency, infections, interstitial lung disease (ILD), airway inflammatory diseases^2,3^. Primary (idiopathic) form of FB is more common in children than in adults^4,5^. The clinical and radiological features of FB are not specific and relatively little is known about the treatment and survival in adults^5^. Here is a review of the clinical, laboratory, radiological and pathological findings, treatment and prognosis of six adult patients with FB identified by surgical lung biopsies (SLB) from Nanjing Drum Tower Hospital in China.

## Subjects and Methods

There were 6076 patients with ILDs admitted to our center between January 2009 and September 2017, and 70 subjects performed surgical lung biopsy (SLB) for diagnosis. Six patients with FB demonstrated by SLB were included in this study. The incidence of FB is about 0.1% in patients with ILD and 8.6% in subjects performed SLB in our single center, respectively. The lung tissue specimens from open lung biopsy (OLB) and video-assisted thoracoscopic surgery (VATS) were reviewed by senior and experienced pathologists. Histological diagnosis of FB required the presence of hyperplastic lymphoid follicles along the distal airways and bronchioles without the evidences of malignant lymphoma^6^. The data of medical records, laboratory examinations, chest high resolution computed tomography (HRCT), pathology and follow up were analyzed retrospectively. All methods were performed in accordance with the relevant guidelines and regulations. Informed consent was obtained from all patients and the study was approved by the ethical committee of Nanjing Drum Tower Hospital. Informed consent—for both study participation, and publication of these indirect identifying identifiers and case details in an online open-access publication (when applicable) was attested.

### Data availability

The data generated during this present study are available from the corresponding author on reasonable request.

## Results

### Baseline clinical characteristics

There were two male and four female patients in our study. The average age was 42 years (range: 31–55 years old). All patients didn’t have any smoking and dust exposure histories. The clinical complains were very mild and often ignored, including cough (3/6), dyspnea (2/6), intermittent lower fever (1/6), and abnormal opacities on chest X-rays by health screening (3/6). Erythrocyte sedimentation rates (ESR) were increased in all six subjects and serum globulin levels were elevated greatly in 5 cases and 4 patients were presented with mild or moderate anemia and elevated serum C reactive protein (CRP). The findings of pulmonary function tests (PFTs) were normal in four cases, one case with mild decreased forced vital capacity (FVC) pred% and two cases with moderate reduced diffusing capacity carbon monoxide (DLCO) pred%. Underlying systemic and pulmonary diseases were present in five cases, including two patients with Sjogren syndrome (SS) and one developed multi-centric castlemans’ disease (MCD), one with MCD, one with IPAF, and one with abscess, respectively. Only one subjects had no underlying systemic diseases or other possible causes, which may be idiopathic (Shown in Table 1).

**Table 1:** Baseline clinical characteristics of 6 Chinese adult patients with FB.

	Case 1	Case 2	Case 3	Case 4	Case 5	Case 6
Sex	M	F	F	F	M	F
Occupation	Clerk	Housekeeper	Bank Clerk	Clerk	Worker	Farmer
Smoking history	No	No	No	No	No	No
Dust exposure history	No	No	No	No	No	No
Age (years old)	33	32	40	55	51	51
Main complaints	None (Health Screening)	Dry cough, dyspnea	Cough, fever, dyspnea	None (Health Screening)	Dry cough	None (Health Screening)
WBC (3.5–9.5 × 10^9^)	6.3	3.9	6.4	4.8	7.7	4.5
Hb (F: 115–150, M:130–175/L)	132	86	86	123	111	81
CRP (0–8 mg/L)	11.3	91.6	68.9	5.1	60.1	56.7
ESR (F < 15, M < 20 mm/h)	85	100	97	28	36	120
LDH (109–245 U/L)	163	220	105	165	166	111
Globulin (20–40 g/L)	54.9	43.8	49.2	35.4	24.6	71.8
IgG (8–16 g/L)	37.5	25.9	30.9	18.0	Not available	44.9
IgA (0.7–3.3 g/L)	7.49	7.6	3.44	1.78	Not available	7.47
IgM (0.5–2.2 g/L)	2.07	1.0	4.74	0.88	Not available	2.84
FVC Pred %	Normal	62.1	Normal	92.6	99.4	105.3
FEV1/ FVC %	Normal	82.20	Normal	89.9	84.5	83.9
DLCO Pred %	Normal	Not available	Normal	77.4	Not available	67.2
Underlying Disease	IPAF	SS	SS, MCD, CLL	—	Lung abscess	MCD

WBC: white blood cell; Hb: hemoglobin; CRP: C reactive protein; ESR: erythrocyte sedimentation rate; LDH: lactate dehydrogenase; FVC: forced vital capacity; FEV1: forced expiratory volume in one second; DLCO: diffusing capacity of the lung for CO2; IPAF: idiopathic pneumonia with autoimmune features; SS: Sjogren syndrome; MCD: multi-centric castlemans’ disease; CLL: chronic lymphocytic leukemia.

### Findings of Chest imaging and Histopathology

Chest HRCT showed relatively nonspecific findings including diffuse interstitial changes, such as nodules, ground glass opacities (GGOs) and interlobular septal thickening, bronchiectasis, cysts, lymphadenopathy, and mass. As shown in Table 2, 5 cases (83.3%) with centrilobular or peribronchiolar nodules and GGOs, 4 cases (66.7%) with interlobular septal thickening, 3 cases (50.0%) with bronchiectasis, 3 cases (50.0%) with cysts, 2 cases (33.3%) with “bud-in-tree”, 3 cases (50.0%) with mediastinal and/or hilar lymphadenopathy were present on chest imaging. Only 1 case (17.6%) showed isolated mass. Diffuse ILD was the main pattern on imaging in these patients with FB (5/6, 83.3%) (Table 2 and Figures).

**Table 2:** Imaging and histological features for 6 Chinese adult patients with FB.

	Case 1	Case 2	Case 3	Case 4	Case 5	Case 6
Main Chest CT Findings	Centrilobular nodules, cysts, diffuse interlobular septum thickening, GGOs	Bronchiolectasis, centrilobular nodules, GGOs, cysts, patchy consolidation	Patchy GGOs, cysts, nodules, interlobular septum thickening, mediastinal lymphadenopath	Centrilobular nodules, bronchiolectasis, GGOs, interlobular septum thickening,mdiastinal and hilar lymphadenopath	Isolated mass, left hilar lymphadenopath	Centrilobular nodules, interlobular septum thickening, GGOs, bronchiolectasis, mediastinal and hilar lymphadenopath
Lung Biopsy	OLB	OLB	VATS	VATS	OLB	VATS
Main Histopathologic findings	FB, peribronchiolar interstitial pneumonitis	FB	FB, mild peribronchiolar interstitial pneumonitis	FB, mild peribronchiolar interstitial pneumonitis	FB, LIP, abscess	FB, Castleman’s disease
Treatment	Prednisone, cyclophosphamide	Prednisone + CTX + hydroxychloroquine	Prednisone	Prednisone	Surgery	None
Follow-up Duration (m)	100	77	66	51	18	15
Outcomes	Alive, stable	Alive, stable	Alive, worse on chest imaging and clinical condition	Alive, recurrent	Alive, stable	Alive, a little worse on imaging, clinical condition stable

GGO: ground glass opacity; OLB: open lung biopsy; VATS: video-assisted thoracoscopic surgery; CTX: cyclophosphamide.

All six patients did SLB, half with OLB and half with VATS. The main pathological manifestation of all subjects was FB, which showed lymphocytic proliferation and lymphoid follicles with reactive germinal centers around the bronchioles, sparing alveolar wall lymphocytic infiltration. Mild peribronchiolar interstitial pneumonitis accompanied with FB were present in 3/6 (50%) cases. One patient was associated with MCD. Lymphocytic interstitial pneumonia (LIP), abscess and FB occurred in the same patient with abscess (Table 2 and Figures).

### Treatment and Prognosis

Four patients were treated with prednisone, two of them used immunosuppressant (cyclophosphamide or hydroxychloroquine) at the same time. Among the other subjects, the patient with lung mass only had surgery, another had only clinical surveillance, without any special treatment. The duration of follow up was form 15 to 100 months (average time: 54.5 months) after diagnosis. At the end of follow up, all subjects were alive. Three patients were stable. One patient was recurrent after prednisone was discontinued; the other one was only a little worse on chest HRCT, while stable on clinical condition; another one developed MCD and became clinically worse than before (Shown in Table 2).

#### Case 1

**Figure 1:**
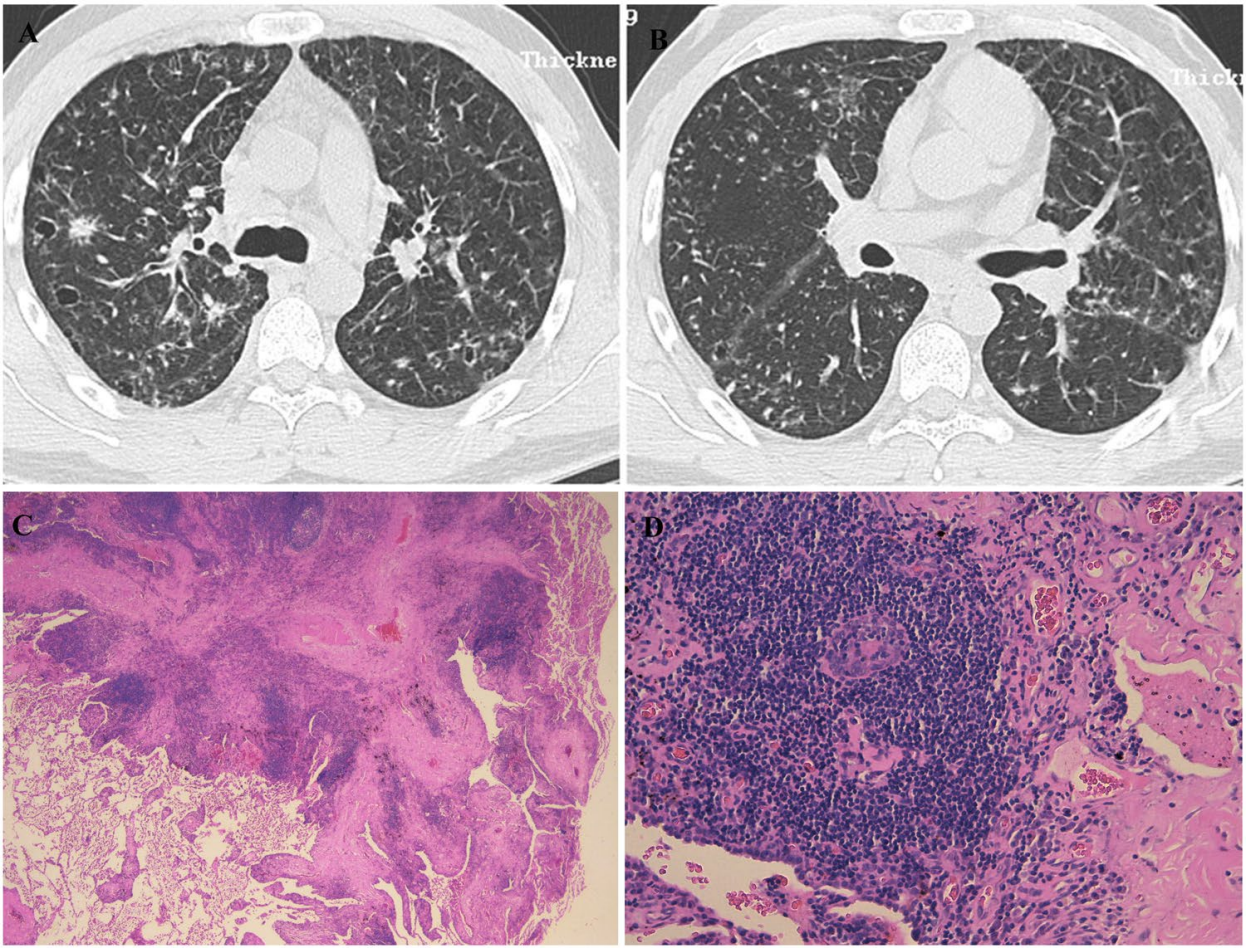
Case 1. (**A**,**B**) Chest HRCT (May 27, 2009) showed bilateral centrilobular nodules (with partial “bud-in-tree” feature), cysts, diffuse interlobular septum thickening and multiple GGOs in some area. (**C**,**D**) Histopathology revealed that FB and peribronchiolar fibrosis, small air way centric lymphoid proliferation with follicular formation and fibrosis reactive germinal center/small mature lymphocytes aggregation (C, HE × 25; D, HE × 200).

A never-smoking 33-year-old male patient was admitted to our hospital because of bilateral multiple nodules on Chest X-ray by healthy screening in May 2009. Chest HRCT showed bilateral centrilobular nodules, cysts, diffuse interlobular septal thickening and GGOs, the nodules were similar to “Tree in bud” in some regions (Fig. 1A,B). PFT was normal. OLB demonstrated FB (Fig. 1C,D). Although the patient had some signs of CTD (serum level of globulin elevated and corneal fluorescent staining positive), it didn’t meet with established classification criteria for a characterisable CTD. It was supposed to be IPAF according the international official statement^7^. He was initially treated with prednisone (0.5 mg/kg/day) and cyclophosphamide (CTX, 400 mg, every 2 weeks), and the dosage of prednisone was tapered gradually. The therapy was continued for 6 months. After six months, the abnormalities on chest CT didn’t have any change compared with before. So prednisone and CTX were stopped. He was free of symptoms and stable on chest imaging during the past 8 years.

#### Case 2

**Figure 2:**
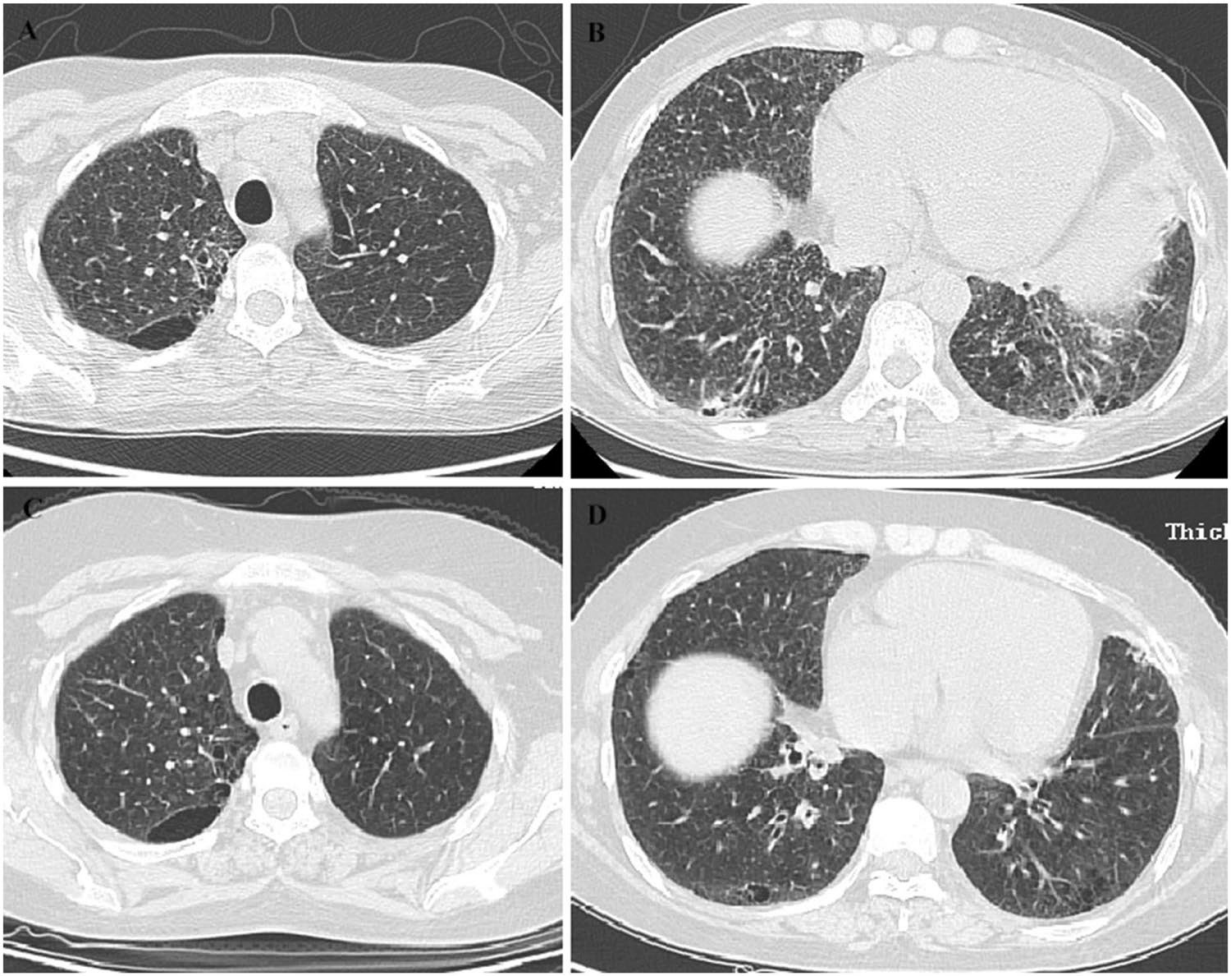
Case 2. (**A**,**B**) Chest CT (Jun 23, 2011) showed centrilobular nodules, patchy consolidation and GGOs, subpleural cysts and bronchiolectasis in both lower lobes. (**C**,**D**) Chest HRCT (Apr 18, 2012) demonstrated that centrilobular nodules, patchy consolidation and GGOs were all absorbed, and the changes of imaging were better than ten months before.

A 32-year-old woman, non-smoker, presenting with dry cough and dyspnea for 5 years was referred to our medical center in June 2011. Chest HRCT showed centrilobular nodules, patchy consolidation, subpleural cysts and bronchiectasis in both lower lobes (Fig. 2A,B). In local hospital, she had done OLB. The histopathology revealed severe chronic inflammation and lymphocyte hyperplasia. By the consultation of the pathologist in our hospital, the changes of histopathology were consistent with FB. She was diagnosed as FB associated with Primary Sjo¨gren’s syndrome (PSS). Prednisone (40 mg/day), CTX (400 mg, per 2 weeks) and hydroxychloroquine were prescribed to her. The symptoms were improved gradually. Ten months later, the lesions of bilateral lungs were less than before on chest CT scan (Fig. 2C,D). Prednisone was tapered to 5 mg/d and CTX was used only one time every three months. The clinical condition was stable during the past 77 months.

#### Case 3

**Figure 3:**
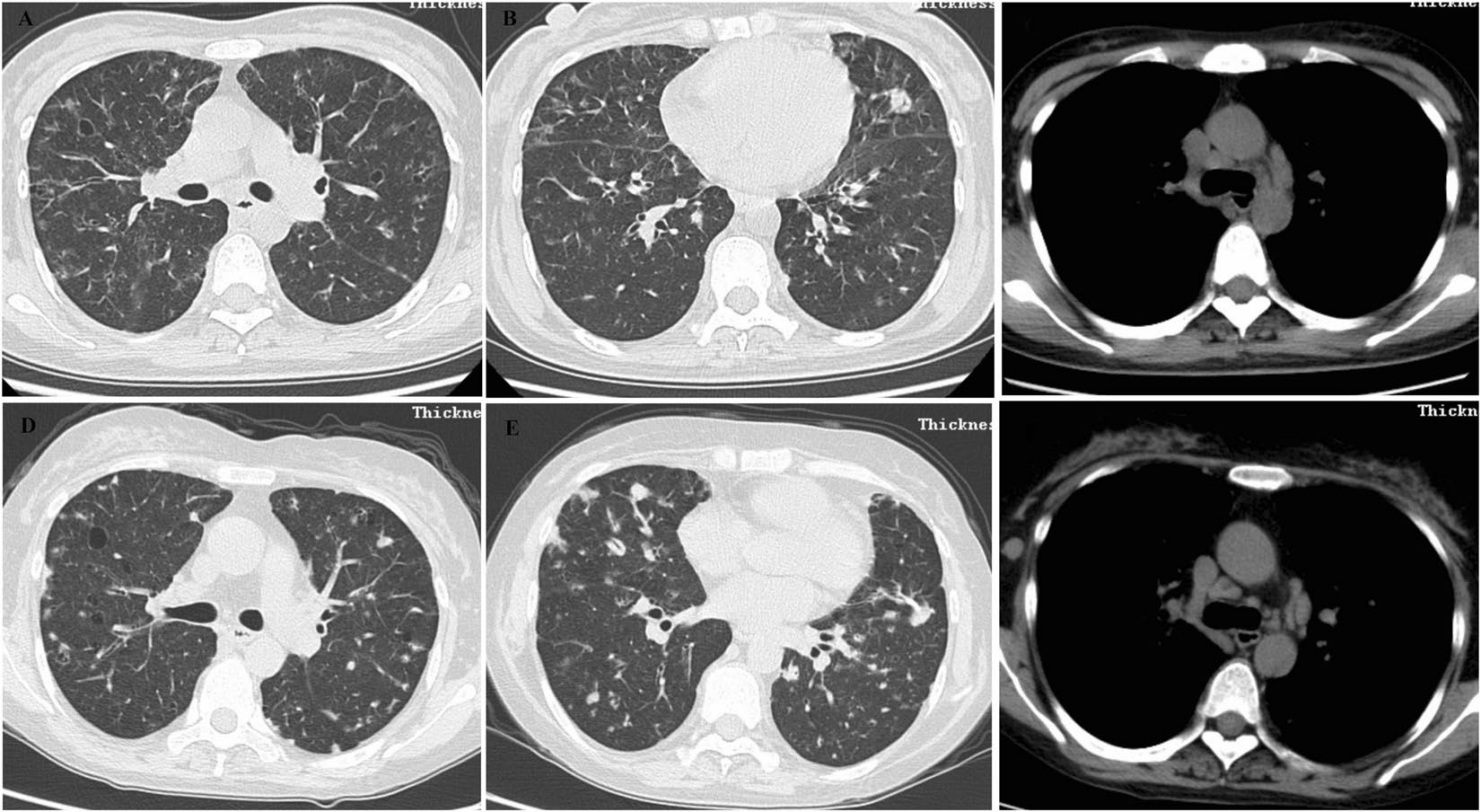
Case 3. (**A**–**C**) Chest CT (Mar 16, 2012) showed nonspecific nodules, interlobular septum thickening, diffuse patchy GGOs on lung window and mediastinal lymphadenopath on mediastinal window. (**D**–**F**) Chest HRCT (May 5, 2016) revealed increased bilateral multiple nodules and cysts, while decreased patchy GGOs compared with before on lung windows. The mediastinal lymphadenopaths were more and larger than before on mediastinal windows.

A never-smoker 40-year-old woman complained recurrent fever for 2 years and productive cough and dyspnea for 3 months and was delivered to our center on March 15th, 2012. She was also complained dry mouth during the past few years. PFT showed there was no ventilation impairment, while DLCO was mildly reduced. Chest HRCT revealed nonspecific nodules, interlobular septal thickening, patchy GGOs and mediastinal lymphadenopathy (Fig. 3A–C). We couldn’t get any useful diagnostic information from the pathology of transbronchial lung biopsy which only showed the changes of chronic inflammation. VATS was performed for large samples of lung tissue. The histopathology revealed typical changes of FB and mild peribronchiolar interstitial pneumonitis. We made the final diagnosis as FB associated with PSS. Prednisone (40 mg/d), CTX and hydroxychloroquine were used for this patient. When prednisone was tapered to less than 15 mg/d, the clinical symptoms were recurrent. So, she had used prednisone 20 mg/d for a long time. On May 5th, 2016, chest HRCT showed bilateral multiple nodules and cysts increased, while patchy GGOs decreased compared with before (Fig. 3D,E), The mediastinal lymphadenopathy were larger than before on mediastinal windows (Fig. 3F). In another hospital, she was diagnosed as MCD associated with chronic lymphocytic leukemia and was treated with chemistry therapy. She planned to do marrow transplantation. The condition was not very well at the end of follow up.

#### Case 4

**Figure 4:**
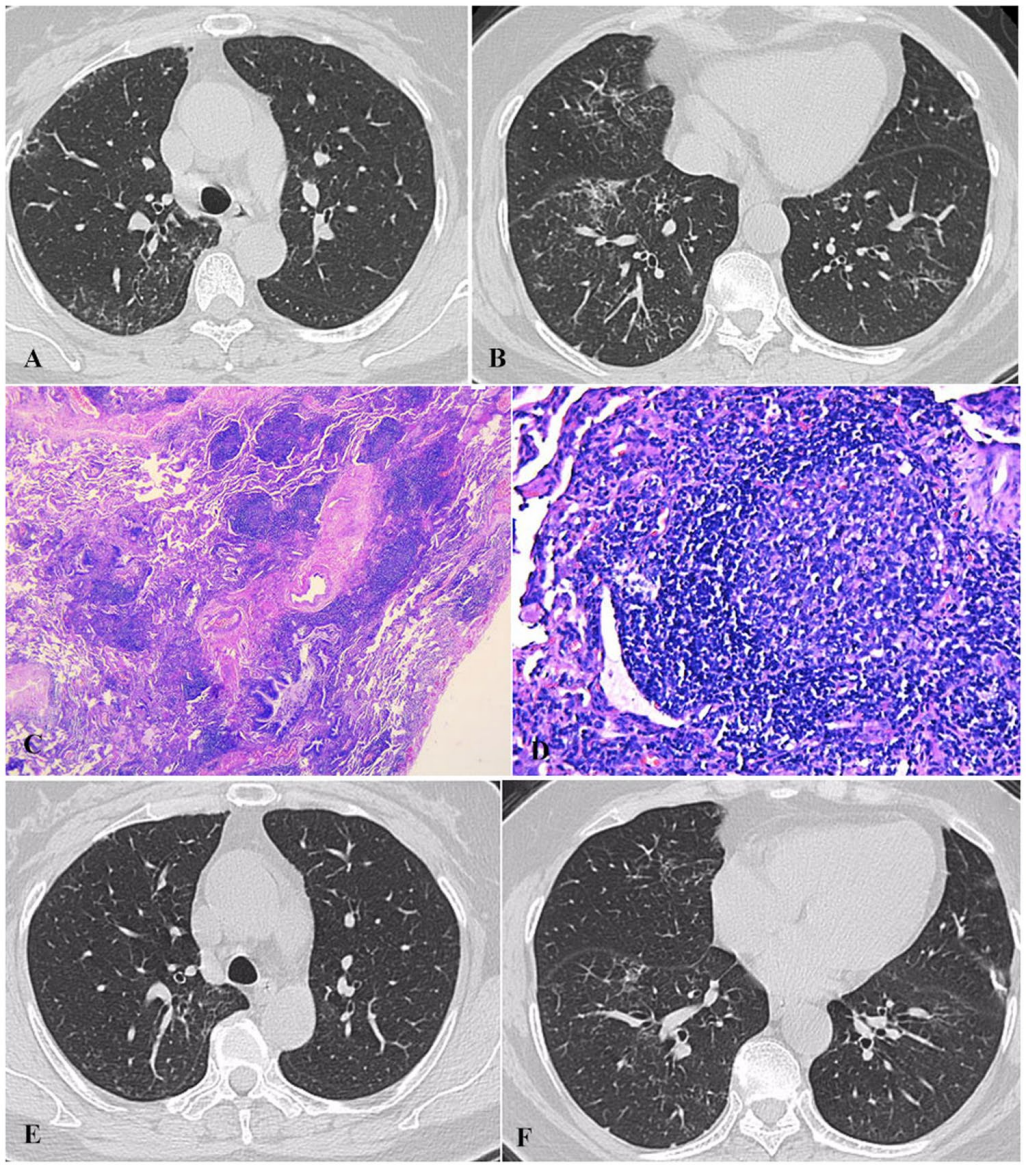
Case 4. (**A**,**B**) Chest HRCT (Jul 3, 2013) showed centrilobular nodules, bronchiolectasis, patchy GGOs, interlobular septum thickening, mediastinal and hilar lymphadenopath. (**C**,**D**) The histopathology of VATS demonstrated FB and ILD and small air way centric lymphoid proliferation with follicular formation and reactive germinal center (C, HE × 25; D, HE × 200). (**E**,**F**) Chest HRCT scans (Nov 25, 2014) revealed that centrilobular nodules, patchy GGOs and interlobular septum thickening were less than before.

A 55-year-old woman, never-smoker, was presented with abnormal opacities on chest imaging by healthy screening on June 3, 2013. She didn’t have any symptoms such as fever, cough, sputum or dyspnea. Chest HRCT showed centrilobular nodules, “Tree in bud”, bronchiectasis, patchy GGOs, interlobular septal thickening, mediastinal and hilar lymphadenopathy (Fig. 4A,B). PFT showed only DLCO pred% was mild decreased. The histopathology of VATS demonstrated FB and interstitial lung disease (Fig. 4C,D). Initially, she was started on prednisone (30 mg/d). The changes on chest HRCT was improved after one year (Fig. 4E,F). Then prednisone was reduced gradually and stopped 2 years later. She had been free of symptoms and didn’t use any medication in the following 2 years. However, the lesions on chest imaging were increased in September 2017, prednisone (30 mg/d) was prescribed to her again.

#### Case 5

**Figure 5:**
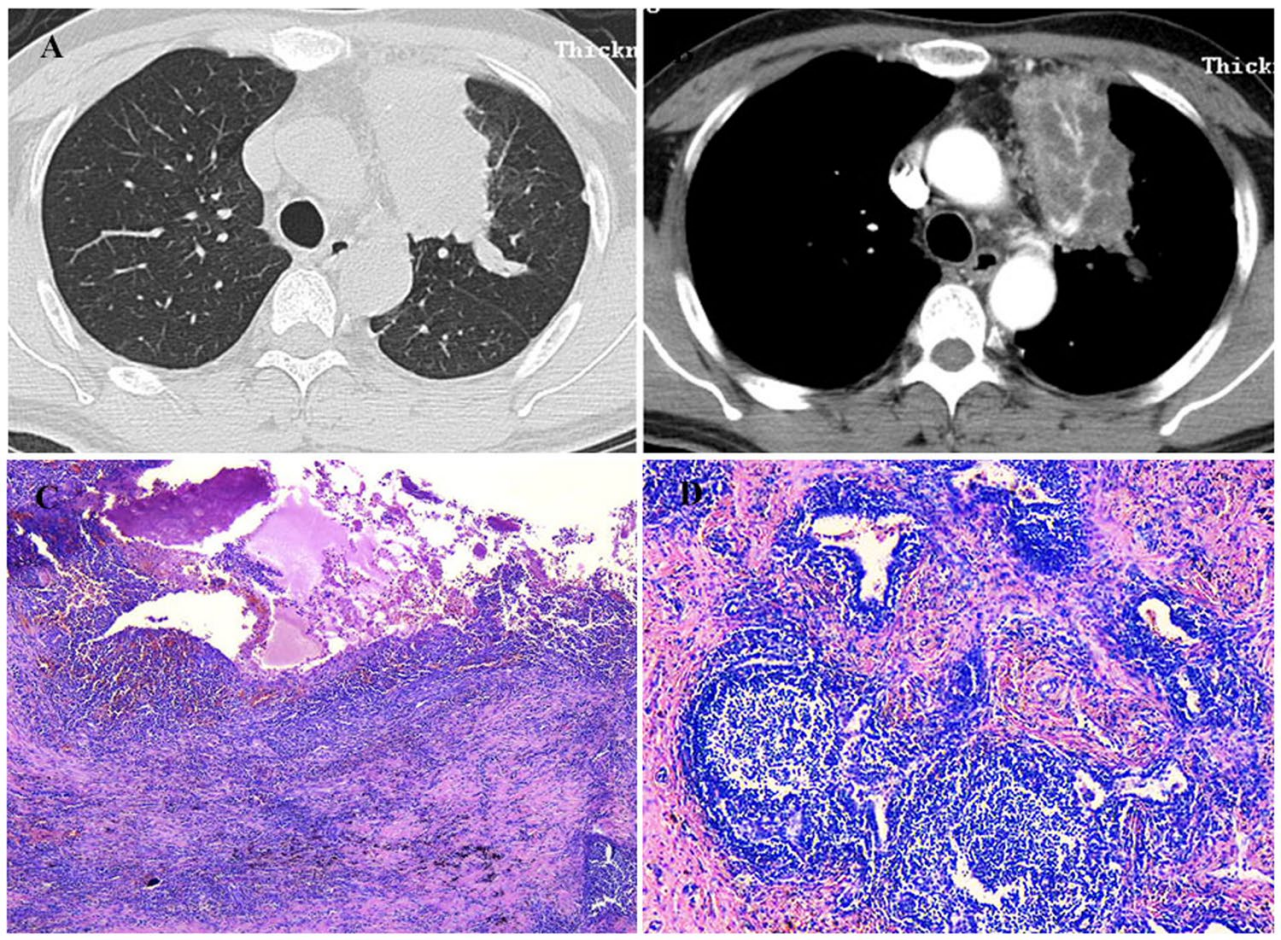
Case 5. (**A**,**B**) Chest HRCT (Feb 24, 2016) showed a mass in the left upper lobe with left hilar lymphadenopathy. (**C**,**D**) The pathology of samples from lobectomy showed FB, LIP and abscess. Microscopic appearance of abscess cavity with chronic inflammatory cells infiltration and fibrosis of cavity wall (C, HE × 25), peripheral region with FB features (D, HE × 100).

A 51-year-old man, non-smoker, was submitted to surgery because of an isolated mass suspected of lung cancer in left upper lobe in March 2016. He had a history of dry cough for more than half a year and expectoration for one month. Chest HRCT showed an isolated mass in the left upper lobe with left hilar lymphadenopathy (Fig. 5A,B). Bronchoscopy showed the complete obstruction of left upper bronchus. The histopathology of transbronchial lung biopsy demonstrated mucosal chronic inflammation. Positron Emission Tomography-Computed Tomography (PET-CT) revealed the consolidation with a little calcium in left upper lung which was regarded as inflammatory mass. He did the lobectomy of left upper lobe. The pathological finding of samples from lobectomy showed FB, lymphocyte interstitial pneumonia (LIP) and abscess. Microscopic appearance showed abscess cavity with chronic inflammatory cells infiltration and fibrosis of cavity wall (Fig. 5C,D). The pathological staining for bacteria, mycobacterium tuberculosis and fungus were all negative. He didn’t have any complaints after the operation for 18 months.

#### Case 6

**Figure 6:**
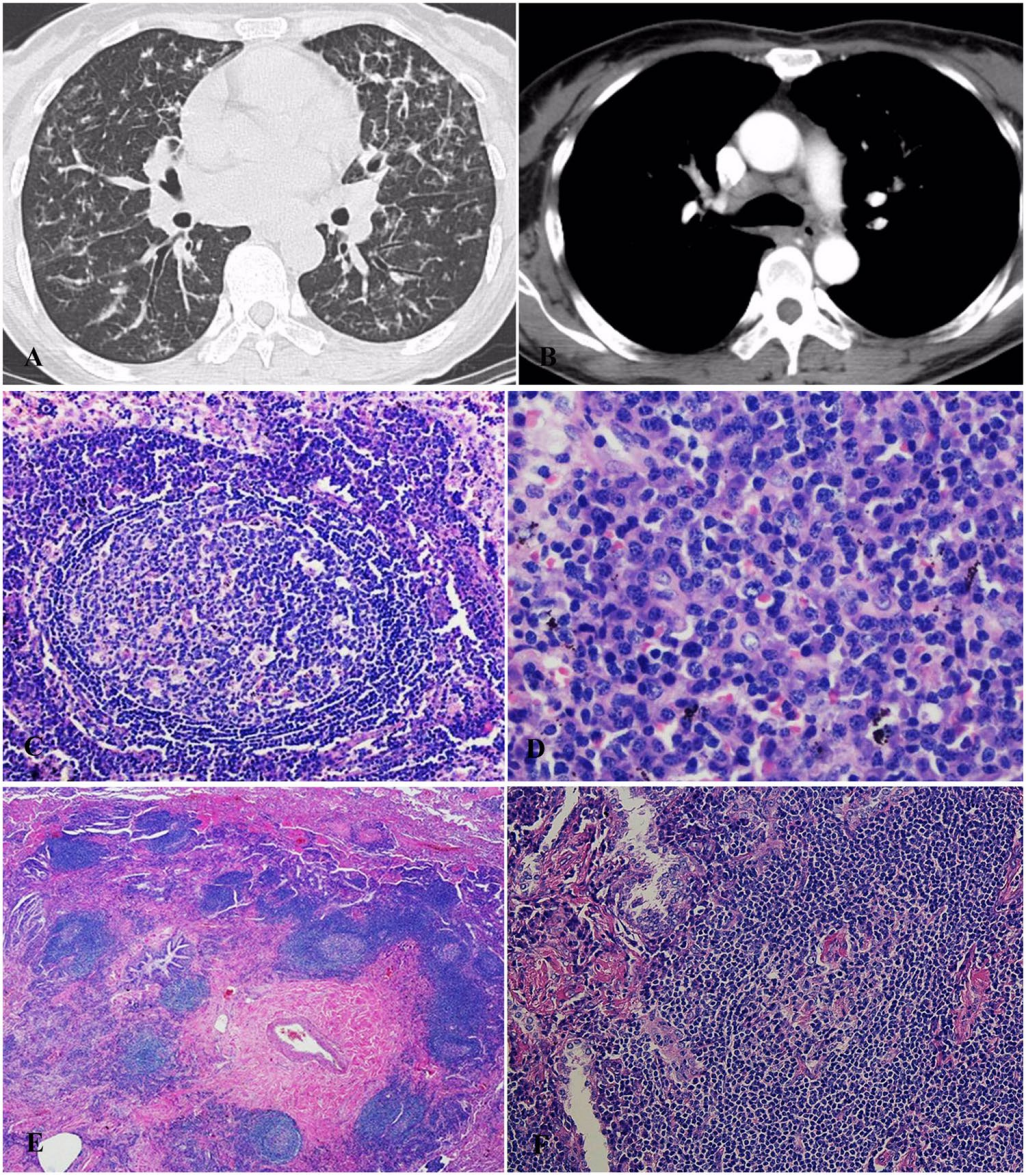
Case 6. (**A**,**B**) Chest HRCT (May 19, 2016) showed that centrilobular and peribronchial nodules, bilateral diffuse thickening of interlobular septum, GGOs, bronchioectasis, multiple mediastinum lymphadenopathy. (**C**–**F**) The pathology of VATS was consistent with FB and MCD. The pathology showed lymphoid proliferation with prominent plasma cells infiltration ‘onion-skin’ pattern with CD features in mediastinal lymphonodes (C, HE × 100; D, HE × 400), bronchiocentric lymphoid proliferation, fibrosis with small pulmonary artery adventitial thickening of collegen in lower power (E, HE × 25), ‘onion-skin’ pattern in higher power with CD features (F, HE × 200). The immumohistochemical staining showed both HHV8 and IgG4 were negative (not showed).

A 51-year-old female patient was presented with abnormal opacities on chest-X ray by health screening on May 17, 2016. No wheezes or crackles were heard on bilateral lungs. Laboratory findings revealed anemia with hemoglobin 86 g/l (normal range: 115–150 g/l) and high serum level of globulin with 71.8 g/l (normal range: 20–40 g/l). However, polyclonal immunoglobulin bands were presented in immunofixation electrophoresis and the marrow biopsy demonstrated the changes of neutrophil, blood platelet and hemoglobin were all hyperplastic, without any evidence of malignancy. PFT showed ventilation function was normal and DLCO Pred % was mildly reduced (67.2%). Chest HRCT scan showed that centrilobular and peribronchial nodules, bilateral diffuse thickening of interlobular septum, patchy GGOs, bronchiectasis, and multiple mediastinal lymphadenopathy (Fig. 6A,B). The pathology of VATS was consistent with FB and MCD (Fig. 6C,D). Because she didn’t have any complaints, we just monitored her. Although the chest CT scan was a little worse after 6 months, the patient didn’t complain of additional symptoms during the past 15 months.

## Discussion

Follicular bronchiolitis was defined by Bienenstock in 1973, who detailed that stimulation of the bronchus-associated lymphoid tissue results in a polyclonal hyperplasia of lymphoid follicles along the bronchioles^6^. FB is an rare lymphoproliferative lung disease and a histopathological diagnosis by surgical or transbronchial biopsy^3^. All patients in current study had the definite diagnosis by surgical lung biopsy. Most cases (5/6) had underlying diseases. The incidence of FB is about 0.1% in patients with ILD and 8.6% in subjects performed SLB in our single center, respectively.

The cause of FB is still unclear. Some authors suggested that it may be generated from a hypersensitivity phenomenon to an unknown antigen or related to a protracted infection^8^. Schmitt *et al*. proposed that Treg cells restrain autoimmune responses, resulting in an organized and controlled chronic pathological process rather than a progressive disease in a new model of autoimmune FB driven by a steady production of antigen-specific T cells^9^. Bezrodnik showed that FB may be a phenotype associated CD25 deficiency presenting homozygous missense mutation in the IL-2 receptor (CD25αR) in a female Argentine patient^10^.

The average age of our six adult patients was 42 years old (range, 31–55 years old). Five cases had underlying diseases and were secondary form, the other one may be idiopathic form. Secondary FB may occur at any age, while the idiopathic form is more commonly seen in pediatric patients^5,11,12^. Yousem SA *et al*. indicated that in the setting of CTD, FB occurred mostly in adults, with a mean age of 44 years, while in patients who were immunocompromised, FB tended to occur at a younger age^8^. FB is just a pathological diagnosis that may be associated with autoimmune diseases, immunodeficiency, infections or obstructive airway diseases^4,13–19^. Less frequently it can be idiopathic, especially in adults. Most cases in our study had underlying conditions, only one case was idiopathic. So, FB in adults may be more common in middle age and associated with multiple systemic or pulmonary disorders. It is important to identify the underlying diseases for those with pathological FB due to different treatment modalities and prognosis.

FB patients are usually present with nonspecific signs and symptoms such as cough, dyspnea, fever, hemoptysis, chest pain, weight loss and crackles on auscultation^13,20^. The clinical symptoms of our six patients were very mild and similar to the published reports, and half cases had no complains. The changes of PFTs were often very mild although the abnormalities on chest imaging were very severe in some cases^13,20^ ESR is a nonspecific biomarker for acute stages in many diseases, but it was elevated in all six subjects. It may be an interesting phenomenon, but the causes need to be investigated further. Cases with underlying CTDs and MCD could show increased CRP, serum globulin and IgG and anemia, just as our cases^15,21,22^. However, serum globulin and IgG were decreased greatly in immunodeficiency patients^18^. Patients with FB usually have mild clinical symptoms and changes in PFTs, the laboratory findings vary greatly depending on the basic clinical settings.

The cardinal imaging features of FB with ILD pattern consist of small centrilobular nodules (1–3 mm), variably peribronchial nodules, and bilateral patchy GGOs^1^. Howling *et al*. suggested that nodules were the most common findings in all 12 patients, Ranging from 1–12 mm in size, which were predominantly centrilobular, some were peribronchial^1^. In our study, patients with ILD had bilateral centrilobular or peribronchial nodules with GGOs. Sometimes nodules can mimic “Tree-in-bud”. Two cases had the classic “Tree in bud”, which may been resulted from the lymphoid follicles becoming densely concentrated in the interstitium adjacent to the bronchioles^1,15^. Cysts, interlobular septal thickening and bronchiectasis were found in our cases. We speculated that cysts were associated with lymphocytic proliferation just as LIP on chest imaging. Interlobular septal thickening may be due to inflammatory cell infiltration in the interstitium on histology^1^. Bronchiectasis was a secondary manifestation of FB, which may be due to the compression of bronchiolar lumen by peribronchiolar infiltration. Mediastinal and/or hilar lymphadenopathy on chest imaging were also seen in some of our cases, which could be related to the underlying diseases, such as MCD, PSS and CTD. Infections and small airway diseases are accompanied by the changes of pathological FB^2,23,24^. The infections of legionella pneumophilia and mycobacterium avium complex could give rise to the development of FB^23,25^. Ikeri, *et al*. reported a child FB patient with collapse consolidation of upper left lobe and prominent air bronchograms had left upper lobectomy on account of recurrent cough and progressive shortness of breath, which was very similar to case 6 with the isolated mass of upper left lobe in our study^26^.

Histological characteristics of FB were hyperplastic lymphoid follicles with reactive germinal centers distributed along bronchovascular bundles associated with mild degrees of lymphocytic and monocytic cell infiltration in the interlobular septum^13^. Six subjects in our study were demonstrated FB on histopathology by SLB. The diagnosis of FB required the presence of hyperplastic lymphoid follicles limited to distal cartilaginous airways without histological or immunophenotypic evidence of malignant lymphoma. The main differentiation of FB is with LIP, which shows several histological features that are similar to those seen in FB, and is also seen in patients with CTDs. Distinction between LIP and FB is based mainly on the extent of lymphocytic infiltration, predominantly peribronchial and peribronchiolar in FB but diffuse in LIP^1^. However, the two conditions can be overlapping just like our one case because they belong to the same spectrum of benign lymphoproliferative lung disease^4^. A diagnosis of FB can be made when the mononuclear cell infiltration has a predominantly peribronchial and peribronchiolar distribution. This distribution and the focal nature of the infiltrates account for the centrilobular and peribronchial distribution of nodules seen on chest imaging.

Management for FB is usually aimed at the underlying diseases. FB associated with CTDs in adults appeared to partially respond to corticosteroid and immunosuppressant therapy^3,13^. Iyonaga *et al*. also showed successful treatment with corticosteroid and cyclophosphamide for 4 year remission in an FB patient related to MCD^16^. However, Hwangbo *et al*. described a case of MCD with FB didn’t have any improvement after the therapy of steroid and azathioprine^21^. Goksel *et al*. reported FB associated with RA that was controlled successfully by colchicine after she did not respond to systemic steroid therapy^2^. However, Kinane *et al*. reported that the response to corticosteroid therapy was minimal in 5 pediatric patients with idiopathich FB^5^. For those with immunodeficiency, Shipe R *et al*. suggested that human immunodeficiency virus (HIV) associated FB has been shown to improve with the anti-retroviral therapy^17^. However, Camarasa EA *et al*. implied that the lung lesions of FB could progress in spite of proper treatment with immunoglobulins, even though the treatment decreased the frequency and severity of infections^18^. In our study, four cases were treated with prednisone in combination with CTX or hydroxychloroquine. The responses to steroids and immunosuppressant were similar to published reports^3,13^, one case progressed, one case was recurrent and another one didn’t response to steroid. Aerni MR reported that one patient was treated with macrolide and then improved, but the role of macrolide therapy for FB need to be explored further^13^.

The prognosis of FB patients are different between the adults and children. Most of FB patients in adults have underlying diseases and the clinical outcomes are usually associated with the underlying conditions^1^. Romero S *et al*. reported that 6 adult patients with FB survived for a mean follow up of 25 months^20^. Aerni MR. *et al*. reported 12 FB cases in adults, there was no deaths related to FB or progressive respiratory disease during a median follow-up period of 47 months^13^. The time of follow up was not very long. Bates CA *et al*. reported that the younger patients with immunodeficiency may develop a more progressive disease^3,27^. Idiopathic FB is more common in children. Kinane BT *et al*. reported 5 pediatric patients with idiopathic FB who were followed up for 2–15 years and the conditions of all subjects improved about 2–4 years of age^5^. Dias A. *et al*. reported a child FB with atelectasis was kept asymptomatic for 9 years on daily inhaled corticosteroid after the lobectomy of left upper lung^28^. FB patients in children reported by Benesch M and Dai YW were all in good conditions for more than 2 years after treatment with steroids or immunosuppressive therapy^11,12^. Up to the end of follow-up in current study, two cases were stable on clinical conditions and chest imaging; one had recurrence and anther one was worse. Patient with abscess associated FB was stable after operation. The Chest HRCT of the patient with MCD associated FB was a little worse after 15 months, but the clinical condition didn’t deteriorate at all. Therefore, the clinical outcomes of adult patients with FB are up to the underlying conditions and relatively favorable. From all of above, we infer that the prognosis of child FB may be better than adult FB.

## Conclusion

Adult FB is associated with several clinical disorders. Patients usually have mild clinical symptoms, elevated ESR and serum globulin, anemia and ILD. The imaging features of FB with ILD are centrilobular or peribronchiolar nodules associated with patchy GGOs. The definite diagnosis needs SBL. Part of patients will respond to corticosteroid therapy. The clinical outcomes of patients with FB in adults depend on their underlying conditions.
